# RATES OF HOSPITALIZATION AND EMERGENCY DEPARTMENT VISITS AND THE QUALITY OF NURSING HOMES IN THE U.S.

**DOI:** 10.1093/geroni/igz038.2536

**Published:** 2019-11-08

**Authors:** Ian Breunig, Qing Zheng, Alan White, Christianna Williams, Allison Muma

**Affiliations:** Abt Associates, Inc., Durham, North Carolina, United States

## Abstract

CMS strives to reduce costs and improve care for nursing home (NH) residents by reducing acute care transfers. We used a national database of Medicare claims and the Minimum Data Set to build NH stays from July 2017 through June 2018 and identify dates of hospital admissions and emergency department visits without hospitalization (ED) among all residents. We calculated rates of 30-day re-hospitalization and ED among short-stay (rehabilitation) residents, and the number of hospitalizations or ED per long-stay resident day (LSRD), then examined associations with NH Five-Star ratings (data.medicare.gov) and other provider characteristics available from Medicare administrative data. We identified 1.79 million short-stays and 898,290 long-stays at 15,576 NHs. Nationally, the 30-day re-hospitalization rate is 22.6%, the short-stay ED rate is 12.0%, there was one hospitalization every 561 LSRD (1.8 per 1000 LSRD), and there was one ED every 617 LSRD (1.6 per 1000 LSRD). Median facility rates were 22.3% (IQR=17.8%, 27.1%) for 30-day re-hospitalizations, 12.0% (IQR=8.7%, 16.1%) for short-stay EDs, 1.6 hospitalizations per 1000 LSRD (IQR=1.1, 2.3), and 1.4 ED per 1000 LSRD (IQR =0.9, 2.2). Higher rates were strongly associated with lower Five-Star ratings, particularly staffing ratings, and larger, for-profit, non-hospital facilities; even after risk-adjustment. NH variation and associations with provider characteristics suggest it is possible to further reduce acute care transfers. CMS incorporated these measures into the Five-Star rating system, providing greater transparency for residents and possibly incentivizing NHs to improve through competition. Future research should monitor success or identify the need for other avenues to improve.

